# On the application of the expected log-likelihood gain to decision making in molecular replacement

**DOI:** 10.1107/S2059798318004357

**Published:** 2018-04-04

**Authors:** Robert D. Oeffner, Pavel V. Afonine, Claudia Millán, Massimo Sammito, Isabel Usón, Randy J. Read, Airlie J. McCoy

**Affiliations:** a Department of Haematology, Cambridge Institute for Medical Research, University of Cambridge, Hills Road, Cambridge CB2 0XY, England; b Lawrence Berkeley National Laboratory, One Cyclotron Road, BLDG 64R0121, Berkeley, CA 94720, USA; c Department of Physics and International Centre for Quantum and Molecular Structures, Shanghai University, Shanghai 200444, People’s Republic of China; d Crystallographic Methods, Institute of Molecular Biology of Barcelona (IBMB–CSIC), Barcelona Science Park, Helix Building, Baldiri Reixac 15, 08028 Barcelona, Spain; e Institució Catalana de Recerca i Estudis Avançats (ICREA), Passeig Lluís Companys 23, 08003 Barcelona, Spain

**Keywords:** maximum likelihood, molecular replacement, *Phaser*, log-likelihood gain, eLLG, LLGI

## Abstract

Molecular-replacement phasing of macromolecular crystal structures is often fast, but if a molecular-replacement solution is not immediately obtained the crystallographer must judge whether to pursue molecular replacement or to attempt experimental phasing as the quickest path to structure solution. The introduction of the expected log-likelihood gain [eLLG; McCoy *et al.* (2017 ▸), *Proc. Natl Acad. Sci. USA*, **114**, 3637–3641] has given the crystallographer a powerful new tool to aid in making this decision. The eLLG is the log-likelihood gain on intensity [LLGI; Read & McCoy (2016 ▸), *Acta Cryst.* D**72**, 375–387] expected from a correctly placed model. It is calculated as a sum over the reflections of a function dependent on the fraction of the scattering for which the model accounts, the estimated model coordinate error and the measurement errors in the data. It is shown how the eLLG may be used to answer the question ‘can I solve my structure by molecular replacement?’. However, this is only the most obvious of the applications of the eLLG. It is also discussed how the eLLG may be used to determine the search order and minimal data requirements for obtaining a molecular-replacement solution using a given model, and for decision making in fragment-based molecular replacement, single-atom molecular replacement and likelihood-guided model pruning.

## Abstract 2

The expected log-likelihood gain can be used to predict the outcome of molecular replacement and optimize molecular-replacement strategies.

## 1. Introduction   

Solving the phase problem by molecular replacement is a problem of signal to noise; the signal for the correct placement of the model must be found amongst the noise of incorrect placements. The signal of a placement is indicated by its translation-function *Z*-score (TFZ), which is the number of standard deviations over the mean (*Z*-score) for the log-likelihood gain on intensity (LLGI) in the translation function (TF). The most sensitive function for scoring the placements is a maximum-likelihood function based on the Rice distribution (LLGI). For a single acentric reflection, 
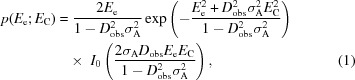
where *E*_C_ is the normalized structure-factor amplitude calculated from the placed model, σ_A_ is the fraction of the calculated structure factor that is correlated with the observed structure factor, and *E*_e_ (the ‘effective *E*’) and *D*_obs_ are derived nontrivially from the observed intensity and its standard deviation (*I*_obs_ and 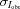, respectively) as described in detail in Read & McCoy (2016 ▸).

The LLGI has a significantly higher signal to noise for molecular replacement than the amplitude-based LLG target (Bricogne & Irwin, 1996 ▸; Murshudov *et al.*, 1997 ▸). The increase is particularly significant when the data are anisotropic and/or strongly modulated owing to the presence of noncrystallographic symmetry, when low-intensity reflections are important for the analysis but reflections with insignificant signal to noise cannot be removed with a simple resolution truncation. The LLGI also allows data beyond the traditional resolution limits to be included in the likelihood calculation, so that all data collected with significant signal to noise, regardless of resolution, can contribute to the signal.

The LLGI required to be confident in a solution for the placement of the first model in molecular replacement depends on the number of parameters that have to be fixed. The results from a database of over 22 000 molecular-replacement calculations, each placing a single model in the asymmetric unit, show that for nonpolar space groups (where the solution has six degrees of freedom) most solutions with an LLGI of 60 or greater are correct, whereas an LLGI of 50 is sufficient for polar space groups and an LLGI of 30 is sufficient for space group *P*1, *i.e.* an LLGI ten times the number of degrees of freedom is sufficient to be confident of success (McCoy *et al.*, 2017). For reference, we call these space-group-dependent LLGI values the solved-LLG values. LLGI values lower than the solved-LLG give proportionately lower confidence in a solution (see Fig. 1 in McCoy *et al.*, 2017 ▸).

Since the value of the LLGI is directly related to the outcome of molecular replacement, the expected value of the LLGI for a correctly placed model for any given molecular-replacement problem will predict the outcome. Following McCoy *et al.* (2017 ▸), the expected value of the LLGI per reflection is a probability-weighted integral over the two unknown parameters *E*_e_ and *E*_C_ of the LLGI, 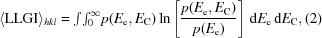
which may be approximated as
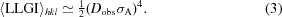
The approximation is particularly good for the low values of *D*_obs_σ_A_ that characterize the cases of most interest, when the signal to noise in the molecular-replacement search is low. The eLLG is the sum for all reflections,
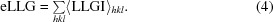
Again following McCoy *et al.* (2017 ▸), the variance of the contribution of one reflection to the eLLG is
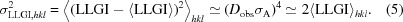
Numerical integrations show that the eLLG for a randomly (incorrectly) placed structure is approximately −eLLG for a correctly placed structure, also with a variance of approximately twice the eLLG. The TFZ for a correct placement is therefore proportional to eLLG^1/2^. This reasoning is consistent with the results of database studies (Oeffner *et al.*, 2013 ▸; McCoy *et al.*, 2017 ▸), where a correct solution is equivalently indicated by a TFZ of 8 and an LLGI of 8^2^ (∼60) in nonpolar space groups, a TFZ value that has long been associated with indicating a correct solution (Table 1 ▸; McCoy *et al.*, 2009 ▸), and a TFZ of 7 and an LLGI of 7^2^ (∼50) in polar space groups.

To calculate the eLLG it is necessary to estimate σ_A_. The resolution-dependent estimates of σ_A_ depend on both the expected coordinate error (Δ_m_) and the expected fraction scattering (*f*_m_) of the model. A Δ_m_ for proteins can be calculated from the sequence identity between the model and the target and the number of residues in the target (Oeffner *et al.*, 2013 ▸), or inferred from other priors. *f*_m_ is deduced by comparing the scattering matter in the model with the expected (ordered) contents of the asymmetric unit. The σ_A_ estimation for eLLG calculation in *Phaser* is given by
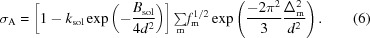
The dependence of σ_A_ on the solvent term in square brackets in (6) is the square of the solvent term previously described (Read, 2001 ▸; McCoy *et al.*, 2017 ▸), after studies indicated better σ_A_ estimation using this functional form (data not shown).

These relationships between *f*_m_, Δ_m_, the number of reflections and the eLLG give fresh insights into molecular replace­ment. Previously, we showed that the eLLG predicted the success of single-atom molecular replacement, which was borne out in the solution of the 1.39 Å resolution structure of residues 22–95 of Shisa3 (McCoy *et al.*, 2017 ▸). We here show how the eLLG can be used more generally to optimize molecular-replacement strategies. Most obviously, the eLLG can be used to predict the outcome of molecular replacement with a model or set of models. We also discuss here the application to decisions regarding minimal data requirements, the burgeoning field of fragment-based molecular replacement, and likelihood-guided model pruning.

## 2. *Phaser* implementation   

The applications discussed below are implemented from *Phaser*-2.8. *Phaser* is distributed through the *CCP*4 (Winn *et al.*, 2011 ▸) and *PHENIX* (Adams *et al.*, 2010 ▸) software suites. The functionality associated with the eLLG is available from the MR_AUTO, MR_ELLG and PRUNE modes of *Phaser*, either from the command line or from the Python interface (see the *Phaser* documentation; McCoy *et al.*, 2009). All functionality can be imported to Python *via* Boost.Python (Abrahams & Grosse-Kunstleve, 2003 ▸). Details of the implementation of each eLLG-based functionality described in the sections below are given in the relevant section.

## 3. Can I solve my structure by molecular replacement?   

If the eLLG for placing a model in the asymmetric unit is well over the solved-LLG then structure solution is likely to be straightforward: high signal to noise and an unambiguous solution.

If the eLLG for placing a model in the asymmetric unit is approaching the solved-LLG then the solution will not distinguish itself clearly from noise. Molecular replacement with *Phaser* will generate a list of potential solutions rather than a single (correct) solution. The number of potential solutions will increase as the signal from the molecular replacement decreases. There is a sigmoidal relationship between the LLGI and the chance of a solution being correct (Oeffner *et al.*, 2013 ▸; McCoy *et al.*, 2017 ▸); half of the solutions with an LLGI equal to half of the solved-LLG are correct (Oeffner *et al.*, 2013 ▸; McCoy *et al.*, 2017 ▸). The solution list is likely to contain the correct solution (an enriched list), even though molecular replacement is not conclusive. It may be possible to distinguish the correct molecular-replacement solution in an enriched list by taking each potential solution through to refinement, particularly wide-convergence radius refinement as implemented in *REFMAC* jelly-body refinement (Murshudov *et al.*, 2011 ▸), *phenix.mr_rosetta* (DiMaio *et al.*, 2013 ▸) or *phenix.den_refine* (Schröder *et al.*, 2010 ▸).

When macromolecular entities in the asymmetric unit are represented by separate models, the molecular-replacement solution is built up by sequential addition; the eLLG can be used to predict the success of each step of molecular replace­ment. The molecular-replacement signal is predicted to be clear when the increase in the eLLG for the placement of a model (not necessarily representing the complete asymmetric unit contents) is over the solved-LLG. Note that the eLLG does not increase linearly as copies of a model are added. Rather, the eLLG increases in proportion to *f*_m_^2^; adding a second copy of a model increases the eLLG to four times that of the first alone, so that, for example, the eLLG for a single copy of a model need only be 20 for the eLLG for two copies to be 80, yielding a change of 60 and corresponding to a potentially clear solution.

### 3.1. Implementation   

*Phaser* lists the eLLG for the placement of the first copy of each search model. If models have already been placed in the asymmetric unit then the eLLG for the addition of another copy of each search model is reported.

### 3.2. Example using *ARCIMBOLDO_LITE*   

The crystal structure of the carboxy-terminal domain of human translation initiation factor Eif5 (PDB entry 2iu1) in space group *P*2_1_2_1_2_1_ contains 179 amino acids with 11 helical segments of lengths ranging from seven to 21 amino acids. Diffraction data to 1.7 Å resolution are available (Bieniossek *et al.*, 2006 ▸). In *ARCIMBOLDO_LITE* (Sammito *et al.*, 2015 ▸), two polyalanine helices 14 amino acids in length are sufficient to phase the data after molecular replacement and density modification interspersed with autotracing with *SHELXE* (Usón & Sheldrick, 2018 ▸). Assuming Δ_m_ = 0.2 Å, which is an appropriate value for a 14-residue helix in the context of *ARCIMBOLDO*, the eLLG is 12 for the placement of the first 14-amino-acid helix and increases to 48 upon correct placement of the second helix. In practice, LLGI values of 27 and 89 are obtained, associated with TFZ scores of 5.7 and 9.7 (*cf*. TFZ ≃ LLGI^1/2^).

## 4. Search strategies   

The eLLG calculation accounts for the trade-off between *f*_m_ and Δ_m_, in which small accurate models may give a higher eLLG than larger more inaccurate models. Searching for models in the order of decreasing eLLG should optimize the path to structure solution.

**Figure 1:**
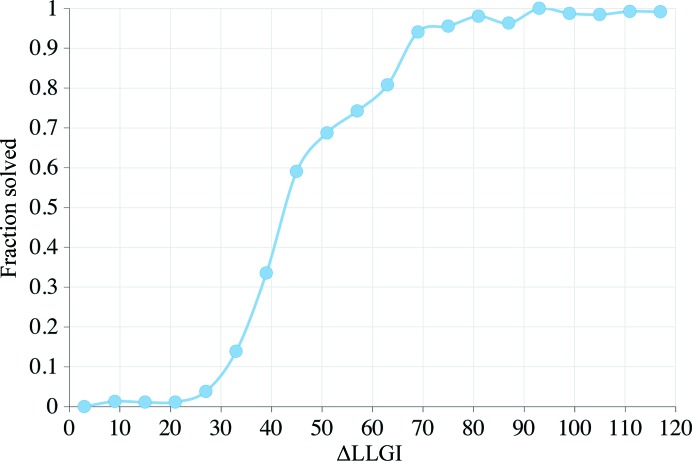
Confidence in the molecular-replacement solution for the placement of two components in the asymmetric unit. The increase in the final refined LLGI score (ΔLLGI = LLGI_2_ − LLGI_1_, where LLGI_1_ is for the placement of the first component and LLGI_2_ is for the placement of both components) provides a clear diagnostic for success in molecular replacement (8762 trials).

When there is more than one model to be placed in the asymmetric unit, search strategies benefit from knowing how many models need to be placed before a clear signal is expected, because if molecular replacement is failing then the search for many copies becomes highly branched and very slow. Using a database of 8762 two-component (heterodimeric) molecular-replacement trials, a clear signal for a correct molecular-replacement solution was found when the gain in the LLGI with the placement of the second component was the solved-LLG (Fig. 1 ▸).

Using the eLLG, molecular replacement can be initiated searching for the number of models for which placement of the last copy should increase the LLGI by the solved-LLG. If the increase in the LLGI reaches the solved-LLG then finding the remaining copies should be straightforward. If the LLGI does not reach the eLLG as expected, further (likely unproductive) search branching is curtailed. If more than one model is available for the target structure then alternative models can be rapidly screened without having to attempt complete structure solution with each.

### 4.1. Implementation   

The default search order for the placement of multiple components in the asymmetric unit is by decreasing order of eLLG. However, if the search for the model with the highest eLLG does not yield a definite solution (implemented in *Phaser* as TFZ > 8) then the search for the first placement is repeated with models for other components of the asymmetric unit until a definite solution is found. If none of the components can be found with a definite solution then molecular replacement continues by building upon the placement of the highest LLGI-scoring first component.

*Phaser* calculates the eLLG for the addition of each model to the current contents of the asymmetric unit during a multi-component molecular-replacement search.

### 4.2. Example   

A mutant form of the four-helix-bundle protein ROP1 was originally solved by an extensive Monte Carlo search for four separate helices (Glykos & Kokkinidis, 2003 ▸). The eLLG values for one, two, three and four helices are shown in Table 2 ▸, and indeed the structure solution becomes straightforward after the placement of the third helix, where the increase in the eLLG is 84.

## 5. Resolution   

**Figure 2:**
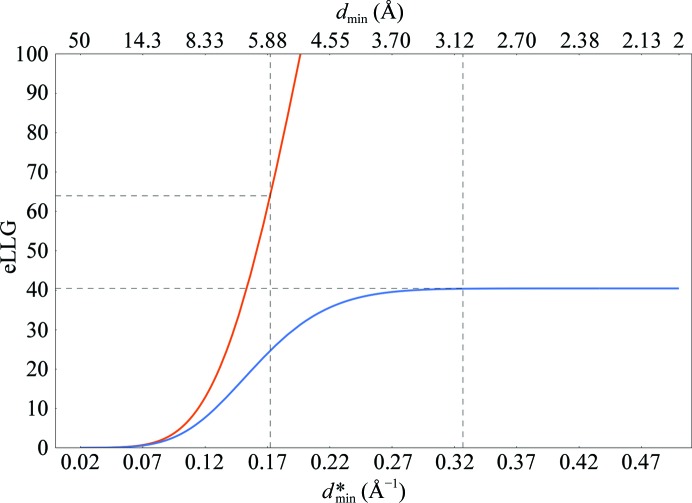
Increase in the eLLG with resolution (orange line) for a model with Δ_m_ = 1.0 Å and a data set with 10 000 reflections to 2.0 Å resolution. An eLLG of 64 (greater than the solved-LLG) is achieved at 5.8 Å resolution. A contrasting case (blue line) shows the increase in the eLLG for a model with Δ_m_ = 1.7 Å. The eLLG will at best be 40.4 (less than the solved-LLG); however, this value is reached asympotically and including data with resolution higher than 3.0 Å (1.8 × Δ_m_) will not increase the eLLG significantly.

At low resolution, where σ_A_ is low owing to errors in modelling solvent and there are fewer reflections in each resolution shell, the eLLG rises slowly as the resolution of the data increases (Fig. 2 ▸). At resolutions where *d* ≫ Δ_m_ each reflection contributes a similar amount to the eLLG, which therefore rises more rapidly with increasing d* (Fig. 2 ▸). At higher resolutions, the contribution to the eLLG from each reflection again drops, and reflections added at resolutions *d* < 1.8 × Δ_m_ do not increase the eLLG significantly (Fig. 2 ▸). An effective eLLG limit is reached asymptotically, with the limit reached in any given case determined by the estimated Δ_m_. This is as expected: the structure-factor contributions from the model are almost uncorrelated with those from the true structure when the Bragg spacing is much less than Δ_m_. For reference, 1.8 × Δ_m_ is called the Δ_m_-limited resolution.

If the data resolution is less than that required to reach the solved-LLG and less than the Δ_m_-limited resolution with any of the available models, molecular replacement is likely to be unsuccessful and therefore should not be pursued at length. The efforts of the crystallographer will be more usefully deployed exploring data-optimization strategies (see, for example, Heras & Martin, 2005 ▸; Alcorn & Juers, 2010 ▸).

Conversely, the eLLG calculated using all of the data may exceed the solved-LLG, in some cases by orders of magnitude. If this is the case then the resolution of the data used for molecular replacement can be cut substantially without jeopardizing a successful outcome. Since the time taken to calculate the LLGI is proportional to the number of reflections, reducing the number of reflections increases the speed of molecular replacement very significantly.

However, in cases where the coordinate error is higher than expected and/or the fraction of the scattering is lower than expected then the LLGI obtained will be lower than the eLLG. If the data do not reach the Δ_m_-limited resolution, truncation of the data using the eLLG will be too severe, leaving too few reflections for successful molecular replacement; molecular replacement must then be repeated with more (all) data included, making the total time for molecular replacement greater than if more (all) data had been used from the outset.

**Figure 3:**
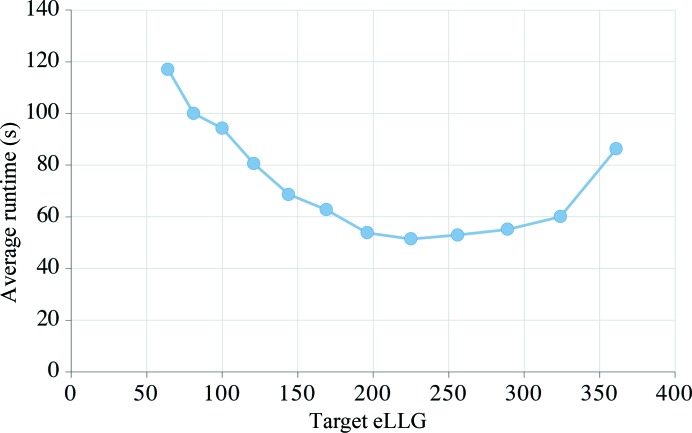
Average *Phaser* runtime for structure solution for 331 successful molecular-replacement test cases *versus* the eLLG used to determine the resolution of the data used for molecular replacement. The optimal-target-eLLG for minimizing the total *Phaser* runtime was 225.

The eLLG used to determine the resolution for data truncation is called the target-eLLG. Rather than using the solved-eLLG as the target-eLLG for data truncation, higher target-eLLG values can be used (which give a higher resolution for data truncation than the solved-eLLG). To optimize the target-eLLG for the total time to solution, a database of 331 molecular-replacement calculations which did not reach the Δ_m_-limited resolution was mined after varying the target-eLLG (Fig. 3 ▸). A target-eLLG of 225, corresponding to a TFZ of 15, optimized the average speed. For reference, we call this the optimal-target-eLLG.

### 5.1. Implementation   

By default, all analyses based on the eLLG are performed with the target-eLLG set to the optimal-target-eLLG. Lower or higher target-eLLG values can be set for any given analysis, but should be greater than the solved-LLG.

In automated molecular replacement, *Phaser* limits the resolution of the data to the resolution required to achieve the target-eLLG (optimal-target-eLLG) and does not include data beyond the Δ_m_-limited resolution. However, the factor of 1.8 applied to Δ_m_ for calculating the Δ_m_-limited resolution is decreased to 1.5 for automated molecular replacement, because refinement of the coordinate error may reduce the coordinate error from the expected value (Δ_m_). If a definite solution (TFZ > 8) is not obtained then the search is repeated using all data.

### 5.2. Example   

Ribosome structures crystallize in large unit cells and so have many more reflections to a given resolution than structures crystallizing in smaller cells. The structure of the hybrid state of the ribosome in complex with the guanosine triphos­phatase release factor 3 (PDB entry 3zvo) can be solved with the 30S (PDB entry 2j00) and 50S (PDB entry 2j01) components of the structure of the *Thermus thermophilus* 70S ribosome complexed with mRNA, tRNA and paromomycin (Selmer *et al.*, 2006 ▸; Jin *et al.*, 2011 ▸). The data extend to 3.6 Å resolution. The coordinate error between the model and the target is predicted to be 0.67 Å (Oeffner *et al.*, 2013 ▸). The percentages of the scattering represented by the 50S and 30S subunits are 45 and 27%, respectively, with one ribosome in the asymmetric unit. The eLLGs for the 50S and 30S components reach the target of 225 at resolutions of 9.2 and 8.1 Å, respectively.

## 6. Fragment-based molecular replacement   

Fragment-based molecular replacement for proteins has its origins in the solution of helical proteins by placing short polyalanine helices (Glykos & Kokkinidis, 2003 ▸; Rodríguez *et al.*, 2009 ▸). A similar method was developed for RNA, using canonical RNA structure motifs to build full solutions (Robertson *et al.*, 2010 ▸). Much recent work has focused on the generation of more general structural fragments, including those from distant homologues (*ARCIMBOLDO_SHREDDER*; Sammito *et al.*, 2014 ▸; Millán *et al.*, 2018 ▸), libraries of structural motifs (*ARCIMBOLDO_BORGES*; Sammito *et al.*, 2013 ▸) or molecular modelling (*AMPLE*; Bibby *et al.*, 2012 ▸). These methods rely on the generation of small but extremely accurate (low coordinate error) fragments, followed by expansion of the placed fragments using aggressive density-modification and model-building methods, such as those implemented in *SHELXE* (Sheldrick, 2010 ▸).

In fragment-based molecular replacement, the coordinate error is not accurately estimated from sequence identity, and so the eLLG cannot accurately estimate the LLGI. However, the eLLG can answer a different question: ‘If the expected coordinate error between my fragment and the structure is a certain value, then what size fragment will I need for successful molecular replacement?’ The fragment library should have fragment sizes tailored to the problem at hand, with an appropriate trade-off between *f*_m_ and Δ_m_ for the data available.

Fragment-based molecular-replacement strategies can be successful even when the eLLG per fragment is much lower than the solved-LLG, and when molecular replacement will only provide an enriched solution list. Strategies to identify the correct solution may include considering the persistence of solutions in solution lists from alternative, but similar, fragments. Key to structure completion in these cases is the application of density-modification, chain-tracing and refinement procedures.

### 6.1. Implementation   

*Phase*r reports the number of polyalanine residues required to reach the target-eLLG (default optimal-target-eLLG) for an input Δ_m_ (or set of input Δ_m_). This number, when calculated in advance of fragment generation, can be used to design bespoke fragment sizes for each molecular-replacement problem.

### 6.2. Example using *ARCIMBOLDO_SHREDDER*   

The structure of the peptidylarginine deaminase from *Porphyromonas gingivalis* (PDB entry 4yt9) contains 432 residues. It can be solved with fragments drawn from a putative arginine deiminase from the same organism (PDB entry 1zbr), sharing 19% sequence identity and a Δ_m_ of 1.5 Å over a core of 231 C^α^ atoms (Millán *et al.*, 2015 ▸). The data in space group *P*2_1_2_1_2_1_ were obtained from a combination of 16 data sets and extended to 1.5 Å resolution. Aiming to find fragments capable of developing into a full solution, Δ_m_ was set to 0.8 Å, so that polyalanine models of 101 residues reached an eLLG of 60. *ARCIMBOLDO_SHREDDER* prepared spheri­cal fragments of PDB entry 1zbr for molecular replacement of 101 residues, and in the course of the *ARCIMBOLDO_SHREDDER* process (Millán *et al.*, 2018 ▸) placed models are given internal degrees of freedom or undergo likelihood-guided pruning (see below) in order to further reduce the Δ_m_ and allow successful density modification and expansion.

## 7. Single-atom molecular replacement   

A single atom is a perfect partial model (Δ_m_ = 0). For such a model, σ_A_^2^ ∝ *f*_m_ and hence LLGI ∝ *f*_m_^2^. Molecular replacement with a single atom, when the structure is large and *f*_m_ is small, requires many reflections because as the number of ordered atoms in the asymmetric unit increases, the LLGI per reflection decreases (∝ *f*_m_^2^) faster than the number of reflections increases for a proportional unit-cell volume (∝ *f*_m_). More reflections may come from higher resolution data or a larger unit cell with the same number of scattering centres (higher solvent content). Since *f*_m_ also depends on the scattering curve, atoms of the same element type but with lower *B* factors will be found with a higher LLGI than those with high *B* factors. Also affecting the scattering factor are the form factors; with regard to protein, S atoms scatter proportionately more at higher resolution than C, N and O atoms. This effect, however, can be negated by a *B* factor raised by as little as 2 Å^2^ above the Wilson *B* factor (Wilson, 1942 ▸). Se atoms in selenomethionine-incorporated proteins are poorer targets for single-atom molecular replacement than their atomic number suggests (*Z* = 34), since selenomethionine residues often display high mobility or disorder (Dauter & Dauter, 1999 ▸).

Single-atom molecular replacement for proteins will be most likely to succeed when the data extend to high resolution, when there is high solvent content and when an S (or heavier) atom is present with a *B* factor lower than the Wilson *B* factor. Direct methods also require high-resolution data (resolved atoms). However, single-atom molecular replacement differs from direct methods in that it does not assume equal atoms, and the likelihood basis for the LLGI inherently takes account of the quality of the available data and the nature of the model. The LLGI for single atoms can reach into double digits in favourable cases. Because of the quadratic dependency of the LLGI on *f*_m_, the placement of as few as two or three single atoms may give an unambiguous substructure. Structure solution can be completed with peak picking from log-likelihood-gradient maps (McCoy *et al.*, 2017 ▸).

### 7.1. Implementation   

For single-atom models, *Phaser* lists the eLLG for the requested search atom type, taking account of the form factors of the atom type relative to the average scattering from protein or nucleic acid, depending on the composition entered. The eLLG is reported for a range of *B* factors downwards from the Wilson *B* factor in steps of 0.5 Å^2^ until the optimal-target-eLLG is reached. This indicates the enrichment that is likely to be obtained by the placement of a first atom that is slightly more ordered than the average atom, and hence how many atoms need to be placed to reach the optimal-target-eLLG.

### 7.2. Example   

**Figure 4:**
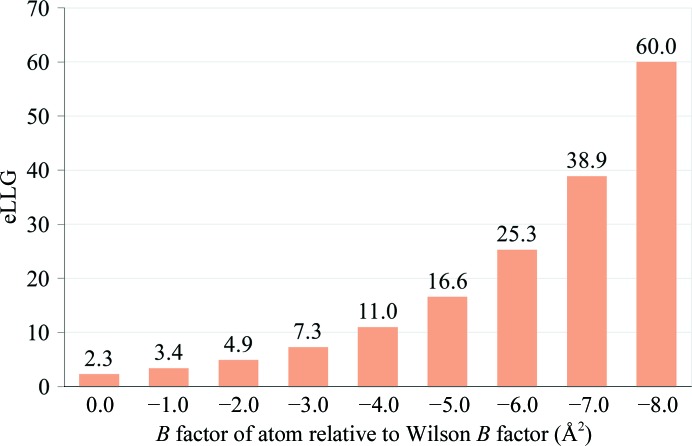
Single-atom molecular replacement for Shisa3 (PDB entry 5m0w) (McCoy *et al.*, 2017 ▸). The eLLG for a single S atom depends on how well ordered it is, as measured by the difference between its *B* factor and the Wilson *B* factor.

The N-terminal domain of mouse Shisa3 (PDB entry 5m0w) can be solved by single-atom molecular replacement (McCoy *et al.*, 2017 ▸). S atoms are the heaviest atoms in the structure, and the eLLG values for S atoms that are more ordered than the Wilson *B* factor are shown in Fig. 4 ▸. The eLLG is 5 for S atoms with a relative Wilson *B* factor of just −2 Å^2^. Seven S atoms were identified by molecular replacement with *Phaser*. Log-likelihood-gradient completion in *Phaser* succeeded in expanding the Shisa3 structure to a total of 56 atoms, mostly well ordered main-chain O and N atoms. The resulting phases were suitable for structure completion through density modification and model building.

## 8. Likelihood-guided pruning   

Editing of structures from the Protein Data Bank prior to molecular replacement is a well established method for improving the signal, and often makes the difference between success and failure (Schwarzenbacher *et al.*, 2004 ▸; Bunkóczi & Read, 2011 ▸; Bunkóczi *et al.*, 2015 ▸). Editing methods range from simple truncation of side chains in the model (polyalanine or polyserine), through the selected removal of atoms based on side-chain substitution, removal of loops and altering *B* factors, to full molecular modelling. At the end of molecular replacement, model editing usually occurs as one of the first steps in structure refinement.

Refinement of atomic occupancies when the phase error is high is not a traditional step during molecular replacement because of the danger of overfitting. The eLLG provides a metric for avoiding overfitting; overfitting is avoided by refining the occupancy of blocks of *n* residues, with *n* determined by the number of residues that give a significant change in the eLLG, *i.e.* the occupancies of *n* residues are constrained to be the same during occupancy refinement. Note that the reduction in the eLLG (ΔeLLG) owing to the removal of *n* residues from a model, where *n* is a small fraction of the total number of residues, is much greater than the eLLG of the placement of the first *n* residues in the asymmetric unit because of the quadratic dependency of the eLLG on the model size. This likelihood-guided pruning is possible for low-resolution data and/or very incomplete models, even when atomic occupancy refinement would not be justified by the data. This includes cases where not all components of the asymmetric unit have (yet) been placed; where multiple copies of a model are present, pruned models can be used as models for the placement of other copies.

The careful parameterization of likelihood-guided pruning can be compared with *B*-factor refinement, which must also be carefully parameterized to account for the amount of data present (Merritt, 2012 ▸). Strategies to constrain *B*-factor refinement include group *B*-factor refinement and TLS refinement (Merritt, 2012 ▸), and are usually chosen heuristically. In likelihood-guided pruning there are no heuristics: the parameterization of the occupancies is directly determined by the data.

Likelihood-guided pruning has two applications. Firstly, the use of likelihood-guided pruning during molecular replacement can relieve packing clashes when the models contain atoms that are outside the true molecular envelope. Secondly, the use of likelihood-guided pruning after molecular replace­ment will accelerate model building and refinement because the process is started from a better model and a better-phased electron-density map.

Likelihood-guided pruning removes atoms that are positioned in solvent regions of the crystal, highly disordered regions of a crystal or regions where the local coordinate error is high. The chemical bonding of atoms is not considered during pruning. Where atoms accurately fill a volume in the crystal pruning will not remove these atoms, even if the placed model does not have the correct atomic types or bonding. This may include cases where the model partly overlies a target and partly overlies a symmetry-related copy of the same target, or partly overlies a different target. Where there is a packing clash between placed models, and more atoms filling a small volume of the asymmetric unit than chemically possible, then likelihood-guided pruning will remove atoms solely on the basis of which ones more accurately represent the true positions of the atoms. It is thus possible that during likelihood-guided pruning the ‘wrong’ residues are removed, where ‘wrong’ can only be defined in the context of *a priori* information that is not available to the pruning algorithm, such as sequence differences between model and target or the likely disorder of residues. Note that similar reasoning could be employed in parameterizing model building and structure refinement more generally.

**Figure 5:**
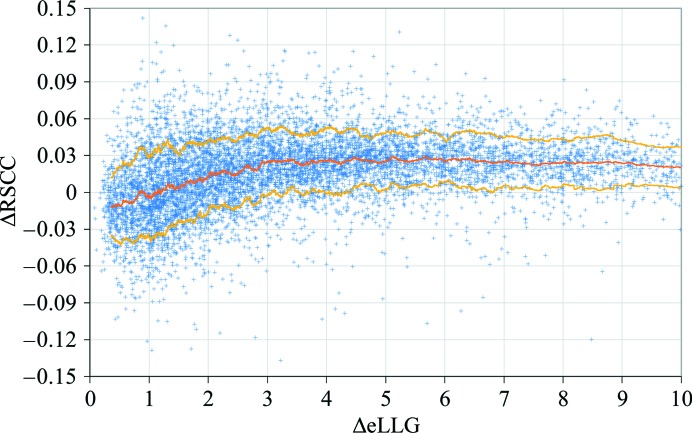
ΔRSCC (7) for 8966 successful molecular-replacement test cases consisting of 1526 targets and between one and 33 models per target (with an average of six models per target). The mean and standard deviation of the distribution of ΔRSCC was calculated in narrow windows of ΔeLLG. The mean (orange line) and one standard deviation either side of the mean (yellow lines) are indicated.

The change in the eLLG for determining *n* (the target-ΔeLLG) was found by probing a database of 8966 molecular-replacement calculations (Oeffner *et al.*, 2013 ▸) for the minimal ΔeLLG that improved the electron-density map without overfitting the data (Fig. 5 ▸). Occupancy refinement was performed in *Phaser* with *n* = 1. The purpose of taking *n* = 1 for the window size was to generate a range of ΔeLLG for the analysis, not to test whether or not *n* = 1 was the appropriate window size; since the model Δ_m_ and the per-residue *f*_m_ were different for each model and target combination, the ΔeLLG was also different for the removal of single residues in each test case. Real-space correlation coefficients (RSCCs) were calculated with respect to the electron density calculated with phases from the refined structure deposited in the PDB (the ‘true’ map), which were assumed to have low phase error. Then,
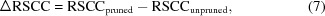
where RSCC_pruned_ is the RSCC between the ‘true’ map and the electron density calculated with phases from the pruned model and RSCC_unpruned_ is the RSCC between the ‘true’ map and the electron density calculated with phases from the unpruned model. Where ΔRSCC was negative, overfitting was indicated. The mean (〈ΔRSCC〉) and standard deviation (σ_Δ__RSCC_) of the distribution of ΔRSCC were calculated in narrow windows of ΔeLLG (Fig. 5 ▸). As expected, 〈ΔRSCC〉 increased with increasing values of ΔeLLG, and
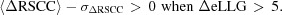



For reference, ΔeLLG = 5 is called the minimal-target-ΔeLLG. Note that this is much lower than the optimal-target-eLLG and indeed the solved-LLG.

### 8.1. Implementation   

Likelihood-guided pruning is currently implemented for protein chains only. When the model is an ensemble of two or more proteins, pruning is performed on the single best model (*i.e.* the model with the lowest Δ_m_). The number of residues *n* to remove to obtain the target-ΔeLLG (by default, the minimal-target-ΔeLLG) is determined. Occupancies are refined in windows of *n* residues for each offset of the window along the protein chain (incremented by single residues). The occupancies of equivalent residues under NCS are not constrained to be the same, because differences in the refined occupancies between NCS copies are valid indicators of differences in crystal packing. The results for each offset of the window are combined by averaging the per-residue occupancy for each offset. This gives the occupancy-refined structure with per-residue occupancies in the range (0.01, 1). The occupancy-refined structure is then converted to a pruned structure, where the occupancies take binary values 0/1 (0 being residues that are pruned) by the application of an occupancy threshold above which the refined occupancies are set to 1 and below which they are set to 0. The optimal threshold is selected by testing thresholds and calculating the LLGI for the model pruned at each value, choosing the threshold generating the highest LLGI. Two coordinate files are output: the pruned structure with occupancies 0/1 and the occupancy-refined structure with occupancies in the range (0.01, 1). The former is ideal for taking forward into model building and refinement, since these expect models with all atoms having occupancy 1. Electron density calculated from the latter may give electron-density maps with lower phase error than those calculated from the former.

As implemented in *Phaser*, the packing test is a pass/fail test based on a pairwise clash score for the trace points [*i.e* approximately 1000 points representing all atoms, C^α^ atoms or a hexagonal grid of points filling the Wang volume (Wang & Janin, 1993 ▸), depending on the protein size (McCoy, 2017 ▸)]. The trace points for the protein are regenerated after likelihood-guided pruning and, since the trace points after likelihood-guided pruning more accurately represent the true atomic volume, solutions with high TFZ discarded for failing the packing test (with the incorrect atomic volume) can be rescued. Likelihood-guided pruning is run by default in the automated molecular-replacement model (MR_AUTO) if the only solution that is obtained has TFZ > 8 but does not pack successfully.

### 8.2. Example   

**Figure 6:**
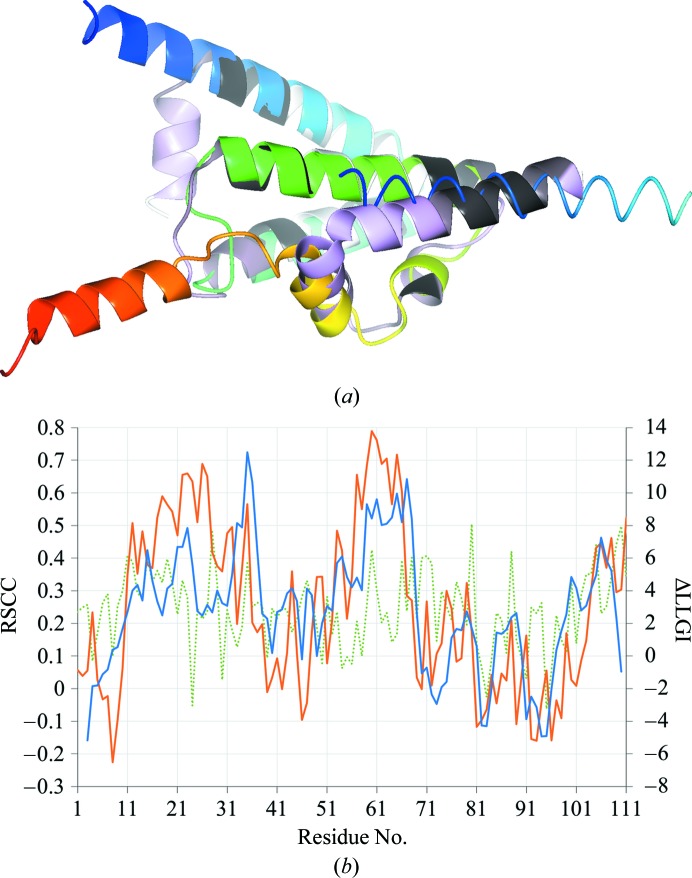
Likelihood-guided occupancy refinement for PDB entry 2hh6 solved by molecular replacement with CASP7 model T0283TS020_2. (*a*) The structure 2hh6 (reference copy, ribbon representation, colour ramp from blue to red from the N-terminus to the C-terminus), the N-terminal helix of a symmetry-related copy of 2hh6 (worm representation, colour ramp as for the reference copy) and the result of likelihood-guided occupancy refinement of the placed model showing occupancies per residue ranging from 1 (black) to 0 (purple). The regions where 2hh6 and the model diverge are the regions where the refined occupancies are close to 0 (the model is shown in purple), and conversely where they coincide the refined occupancies are close to 1 (the model is shown in black). The window size for occupancy refinement was five residues, determined by the optimal-target-ΔeLLG. This figure was produced with *CCP*4*mg* (McNicholas *et al.*, 2011 ▸). (*b*) The difference between the LLGI for the placed model T0283TS020_2 before and after removing five residues centred on each residue along the chain (blue line). The RSCC per residue is shown between the placed model T0283TS020_2 and the ‘true’ map (see text; orange line) and between the placed model T0283TS020_2 and the ‘model’ map (see text; dotted green line). The RSCC of the model to the ‘true’ map is better predicted by the change in LLGI (orange line *versus* blue line) than by the RSCC to the ‘model’ map (orange line *versus* dotted green line).

The structure with PDB code 2hh6 (112 residues), a protein from *Bacillus halodurans* of unknown function, was modelled as part of the seventh Critical Assessment of Techniques for Protein Structure Prediction (CASP7 target T0283). The model T0283TS020_2 and target 2hh6 differ significantly at several places (Fig. 6 ▸ *a*). At the N-terminus, the first 27 residues of 2hh6 form a continuous helix that starts beyond the body of the protein, but in T0283TS020_2 the first two turns of this helix are modelled as a short helix folded back against the body of the protein. At the C-terminus, the last 22 residues of 2hh6 form a loop followed by a three-turn helix, but in T0283TS020_2 these residues are modelled as a shorter loop followed by a five-turn helix which do not overlie 2hh6, and indeed run in the opposite direction away from the true structure (Fig. 6 ▸ *a*). Five residues of T0283TS020_2 represent 4.5% of the total scattering and give ΔeLLG = 4.9. Pruning based on a window size of five residues removes residues at the N-terminus and C-terminus where the model and target diverge (Fig. 6 ▸ *a*). The change in the LLGI owing to the removal of five residues is much more predictive of the model quality along the chain, as judged by the RSCC of the model against the ‘true’ map (defined in §[Sec sec7]7), than is the RSCC between the model and the electron density calculated using phases from the unpruned model (the ‘model’ map; Fig. 6 ▸ *b*). The change in the LLGI is therefore a better indicator of model quality along the chain than the RSCC between the model and the model-phased electron density, as is traditionally used.

### 8.3. Example   

**Figure 7:**
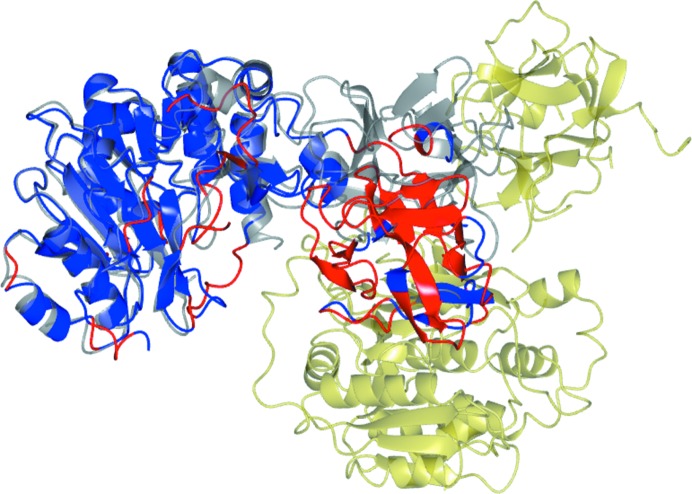
The molecular-replacement solution of PDB entry 1xhb (grey) solved with PDB entry 2d7i (red and blue) after likelihood-guided pruning of the placed 2d7i model, where blue indicates an occupancy of 1 and red indicates an occupancy of 0. The symmetry-related copy of 1xhb that clashes with the model after initial molecular replacement is shown in gold.

A test case using polypeptide α-*N*-acetyl-galactosaminyltransferases shows the use of likelihood-guided pruning to remove packing clashes [target PDB entry 1xhb (Fritz *et al.*, 2004 ▸) and model PDB entry 2d71 (Kubota *et al.*, 2006 ▸)]. The sequence identity between the model and the target is 45%. The transferase structures consist of two domains and these have a different hinge angle in the model and target structures. A model was prepared from PDB entry 2d71 using *Sculptor* (Bunkóczi & Read, 2011 ▸). In the default MR_AUTO mode, *Phaser* finds a solution with high TFZ, but the hinge angle between the domains manifests itself as a clash in the packing of this solution. After automatic likelihood-guided pruning, the majority of the residues in the smaller domain of 2d71 are removed and the pruned model passes the packing test (Fig. 7 ▸).

## 9. Twinning   

**Figure 8:**
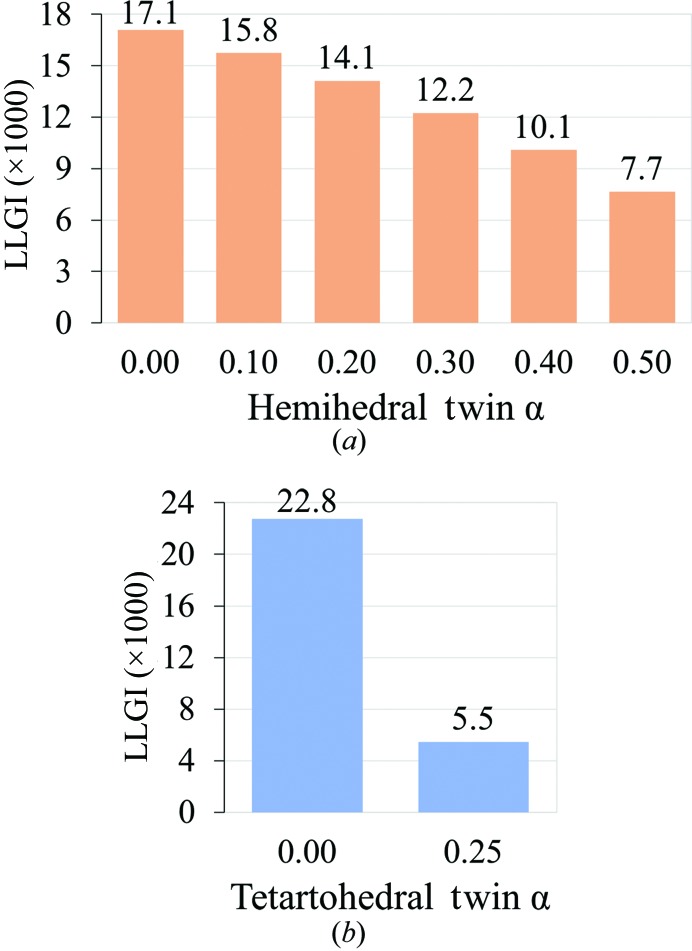
The LLGI as a function of the twin fraction for calculated data, showing that the LLGI of the molecular-replacement solution decreases in proportion to the twin fraction. (*a*) The LLGI as a function of the hemihedral twin fraction for calculated data for the test case of the β-lactamase (BETA)–β-lactamase inhibitor (BLIP) complex (Strynadka *et al.*, 1996 ▸). (*b*) The LLGI as a function of tetartohedral twinning for calculated data for the test case of human complement factor 1 (Roversi *et al.*, 2012 ▸).

Twinning reduces the LLGI, and so a correction term should, in principle, be applied to the eLLG. The reduction in the eLLG was studied for hemihedral and tetartohedral crystal twinning, which are particular cases of (pseudo)merohedral twinning where the number of twinned domains is two and four, respectively. The BETA–BLIP structure (Strynadka *et al.*, 1996 ▸), which has previously been used as a test case for *Phaser* (Storoni *et al.*, 2004 ▸; McCoy *et al.*, 2005 ▸; McCoy, 2007 ▸), was used to generate simulated data with different hemihedral twin fractions, and the LLGI was calculated for the structure given the simulated data (Fig. 8 ▸ *a*). The relationship between the LLGI and the twin fraction is approximately linear for hemihedral twinning, so that a twin fraction of a half leads to a halving of the LLGI for untwinned data. A higher order twinning test was performed with the structure of human complement factor 1 (PDB entry 2xrc), which has *P*1 symmetry and tetartohedral twinning. For perfect tetarto­hedral twinning the degree of reduction in the LLGI was a factor of four (Fig. 8 ▸ *b*).

### 9.1. Implementation   

Since the presence, order and/or fraction of twinning cannot be determined with certainty in advance of structure solution, even if twinning is indicated the eLLG is not decreased. Indeed, other data pathologies, which are often associated with twinning, may make molecular replacement more difficult than expected. If molecular replacement fails with twinned data, it may be helpful to increase the target-eLLG.

## 10. Discussion   

Experienced users of *Phaser* may wish to see a solution with LLGI ≫ 64 and TFZ ≫ 8 to increase the certainty that the solution is correct. While an LLGI > 64 and a TFZ > 8 have been proven to be significant, a target-eLLG of 225, equivalent to TFZ = 15, was found to optimize the time to structure solution. It is likely that the preference of the experienced user for LLGI ≫ 64 and TFZ ≫ 8 is partly informed by their experience of the time taken for structure solution, rather than the outcome. To give the user additional information about the certainty of a solution after automated molecular replace­ment with *Phaser*, a ‘TFZ-equivalent’ is calculated, which is the TFZ that would have been obtained if the refined position were found (*i.e.* located exactly on the search grid) in a translation function performed with the model in the refined orientation, using all data.

Pathologies in the data that violate the assumptions of the likelihood function have a severe impact on the likelihood estimates. The eLLG will be an accurate estimator of the LLGI when data are isotropically distributed with a Wilson distribution. Data anisotropy (Murshudov *et al.*, 1998 ▸) and many forms of translational noncrystallographic symmetry (tNCS) modulations (Read *et al.*, 2013 ▸; Sliwiak *et al.*, 2014 ▸) can be accounted for. However, when the data contain uncorrected pathologies, the use of the eLLG to lower the resolution for molecular replacement may cause solutions to be missed; incorrect placements obtained with the minimal number of reflections that have TFZ > 8 must be avoided with the *Phaser* automated search algorithm, because the placement will be taken to be correct and the search terminated.

The order of the tNCS is not used to increase the *f*_m_ for the eLLG calculation (Read *et al.*, 2013 ▸). By default, *Phaser* places the number of tNCS-related molecules in one step of the rotation and translation functions. The *f*_m_ for a single copy could thus be multiplied by the number of tNCS-related copies in the calculation of the eLLG. The eLLG-truncated resolution will thus be higher than necessary to achieve the eLLG in the presence of tNCS. However, errors in the modelling of the tNCS during the rotation and translation function, particularly when the tNCS relates more than two copies, means that conservative resolution truncation is prudent.

Poor estimates of σ_A_ will degrade the accuracy of the eLLG. Estimates of σ_A_ depend on both Δ_m_ and *f*_m_. The Δ_m_ estimated from the sequence identity between the model and the target and the number of residues in the target (Oeffner *et al.*, 2013 ▸) has an associated error with a fractional standard deviation of 0.2. In the future, it may be possible to incorporate the uncertainty in the Δ_m_ estimation into the eLLG estimate. The eLLG analysis also assumes that the *B* factors of the components are equal to the Wilson *B* factor. Differences between the two manifest as errors in *f*_m_. Uncertainties in Δ_m_ and search *B* factor may be accounted for by performing a grid search over these estimates rather than relying on a single estimate. Note that the input values of Δ_m_ and search *B* factor are only important until a solution is found and retained in the potential solution list, even with low signal to noise, because the Δ_m_ and *B* factor are refined (to optimize the LLGI) at the end of molecular replacement in *Phaser*.

The eLLG only provides a metric for the likely success or failure of molecular replacement. It does not provide a metric for whether or not a molecular-replacement solution can be converted into a completed, validated structure suitable for publication and deposition in the PDB. High-resolution data beyond those required for successful molecular replacement will often be required to reduce model bias. It may be possible to develop other likelihood-based metrics for determining the limits on the structure quality possible with the data available.

Judicious use of the eLLG for decision making in molecular replacement should reduce the time to structure solution in most cases. It should also guide the development of more efficient automated molecular-replacement pipelines, particularly those based on fragment libraries.

## Figures and Tables

**Table 1:** Guidance for the outcome of molecular replacement in *Phaser* for the placement of the first model in nonpolar space groups, showing the relationship between the translation-function *Z*-score TFZ and the LLGI (TFZ ≃ LLGI^1/2^)

Solved?	TFZ	LLGI
No	<5	<25
Unlikely	5–6	25–36
Possibly	6–7	36–49
Probably	7–8	49–64
Definitely†	>8	>64

† TFZ and LLGI are significant at lower values for the first model in polar space groups: TFZ = 7 and LLGI = 50 for the first model in polar space groups, and TFZ = 5.5 and LLGI = 30 for the first model in space group *P*1.

**Table 2:** ROP1 (Glykos & Kokkinidis, 2003 ▸) solved with a 25-residue polyalanine helix The LLGI values achieved in the search follow the eLLG values predicted from an Δ_m_ of 0.3 Å, an appropriate value for a helix of this length, and a scattering fraction of 0.14. The TFZ exceeds 8 for the placement of the third helix, when the increase in the LLGI (ΔLLGI) is 84.

Helix number	eLLG	LLGI	ΔLLGI	TFZ
1	20	63	—	4.5
(1 +) 2	79	113	50	7.0
(1 + 2 +) 3	177	197	84	11.6
(1 + 2 + 3 +) 4	315	281	84	9.9
